# Hypertension, type 2 diabetes, obesity, and p53 mutations negatively correlate with metastatic colorectal cancer patients’ survival

**DOI:** 10.3389/fmed.2023.1091634

**Published:** 2023-01-23

**Authors:** Alessandro Ottaiano, Mariachiara Santorsola, Luisa Circelli, Francesco Perri, Marco Cascella, Francesco Sabbatino, Maurizio Capuozzo, Vincenza Granata, Silvia Zappavigna, Angela Lombardi, Marianna Scrima, Nadia Petrillo, Monica Ianniello, Marika Casillo, Oreste Gualillo, Guglielmo Nasti, Michele Caraglia, Giovanni Savarese

**Affiliations:** ^1^Istituto Nazionale Tumori di Napoli, IRCCS “G. Pascale”, Naples, Italy; ^2^AMES, Centro Polidiagnostico Strumentale srl, Naples, Italy; ^3^Oncology Unit, Department of Medicine, Surgery and Dentistry, University of Salerno, Salerno, Italy; ^4^Coordinamento Farmaceutico, Ercolano, Italy; ^5^Department of Precision Medicine, University of Campania “L. Vanvitelli”, Naples, Italy; ^6^Laboratory of Molecular and Precision Oncology, Biogem Scarl, Institute of Genetic Research, Ariano Irpino, Italy; ^7^Servizo Galego de Saude and Instituto de Investigación Sanitaria de Santiago, Neuroendocrine Interactions in Rheumatology and Inflammatory Diseases, Research Laboratory 9, Santiago University Clinical Hospital, Santiago de Compostela, Spain; ^8^IDIS, Instituto de Investigación Sanitaria de Santiago de Compostela, Grupo C027 NEIRID, Santiago de Compostela, Spain

**Keywords:** hypertension, type 2 diabetes, obesity, p53, prognosis, NGS, metastatic colorectal cancer

## Abstract

**Introduction:**

We studied the predictive and prognostic influences of hypertension (HT), type 2 diabetes (T2D), weight, and *p53* mutations in metastatic colorectal cancer (CRC) patients.

**Patients and methods:**

T2D was diagnosed according to the ADA criteria. HT was classified according to the ACC/AHA guidelines. BMI (body-mass index) was calculated and classified according to the WHO criteria. TruSigt™Oncology 500 kit was applied to construct the genomic libraries for Next Generation Sequencing (NGS) analysis. The Illumina NovaSeq 6000 technological platform and the Illumina TruSight Oncology 500 bioinformatics pipeline were applied to analyze results. Overall survival (OS) was calculated through Kaplan-Meier curves. Univariate and multivariate analyses were performed to assess the relationships between clinical and/or molecular covariates. Associations between HT, T2D, BMI, p53, and clinical variables were evaluated by the χ2 test. *P* < 0.05 were considered statistically significant.

**Results:**

Two-hundred-forty-four patients were enrolled. One-hundred-twenty (49.2%), 110 (45.1%), and 50 (20.5%) patients were affected by overweight, HT, and T2D, respectively. DC (disease control) was achieved more frequently in patients without T2D (83.1%) compared to the diabetic ones (16.9%) (*P* = 0.0246). DC, *KRAS* mutational status, T2D, BMI, and concomitant presence of T2D, BMI, and HT associated with survival (*P* < 0.05). At multivariate analysis, age (≥65 vs. <65 years), response to first-line chemotherapy (DC vs. no DC), and concomitant presence of T2D, BMI, and HT (HR: 4.56; 95% CI: 2.40–8.67; *P* = 0.0217) emerged as independent prognostic variables. *P53* was mutated in 31/53 analyzed cases (60.4%). The most frequent gene variants were p.Arg175His and p.Cys135Tyr. High BMI (>25 kg/m^2^) associated with occurrence of *p53* mutations (*P* < 0.0001). *P53* mutated patients presented a worse prognosis compared to the wild-type ones (HR: 3.21; 95% CI: 1.43–7.23; *P* = 0.0047).

**Conclusion:**

Diabetic, hypertensive and overweight metastatic CRC patients are a negative prognostic subgroup deserving specific therapeutic strategies. *P53* mutations associate with prognosis and BMI unrevealing complex and unexplored connections between metabolism and cancer occurrence.

## Introduction

Hypertension (HT) and type 2 diabetes (T2D) are two common chronic diseases affecting more than five hundred million people worldwide (1, 2). They are profoundly interrelated since HT is more frequent in T2D patients compared with those without T2D. Furthermore, patients with HT develop T2D more frequently than individuals with normal blood pressure (3, 4). The occurrence of obesity, HT, T2D, and dyslipidemia is designated as “metabolic syndrome,” which is one of the major causes of deaths related to heart disease and stroke (5). Colorectal cancer (CRC) is the third most common cancer, and it has been estimated that in 2020, about 1 million people dead for metastatic CRC (mCRC) (6). As for HT and T2D, the large part of CRCs is attributable to incorrect lifestyle. Well-known risk factors are diet high in animal products and low in vegetables and fruits, smoking, low physical activity, vitamin D deficiency, obesity, and helicobacter pylori infection (7–9). Interestingly, about 35% of mCRC patients have T2D and/or HT, and both have been recognized as risk factors for CRC development (10–12).

The etiopathogenesis of T2D relies on insulin resistance and/or reduced secretion from beta-cells (13). The result is a persistent increase of blood glucose levels (hyperglycemia) leading to micro- and macro-vascular generalized damages (nephropathy, neuropathy, maculopathy, central and peripheral vasculopathy) (14). The etiopathogenesis of primary HT include alterations in the activity of sympathetic nervous system, the renin-angiotensin system, the pathways involved in calcium and sodium homeostasis, the structure and reactivity of the vascular smooth muscle network (15–17). However, both T2D and HT are multigenic diseases where several genetic/epigenetic factors (including genetic polymorphisms) may concur to determine heterogeneous phenotypes (18). Interestingly, insulin resistance and activation of renin-angiotensin system are frequently observed in obesity with a body-mass index (BMI) > 30.0 kg/m^2^ (19, 20).

The treatment of mCRC has been enriched in last years by the introduction of biologic drugs in specific clinical settings. They include the anti-angiogenic drugs (bevacizumab, aflibercept), the anti-EGFR (Epidermal Growth Factor Receptor) drugs (panitumumab, cetuximab), a multi-kinases inhibitor (regorafenib), and two immunological check point inhibitors (pembrolizumab and nivolumab). To date, a “continuum of care” approach including both cytotoxic and biologic drugs can prolong the survival of mCRC patients up to 30 months (21). Previous studies have explored the prognostic value of HT in mCRC patients treated with bevacizumab containing chemotherapy (22, 23), as well as the prognostic power of T2D (24) or BMI (25–29) predominantly in early stage CRC.

Furthermore, the genetic background of tumors influences clinical behavior including response to treatments and time-to-outcome extent. The scientific community is committed to study genotype/phenotype relationships in order to identify biomarkers exploitable for diagnostic and/or therapeutic applications. In this context, the extremely pleomorphic and important roles of p53 protein in cancer and host metabolism (30), prompted us to explore the mutational landscape of *p53* in our patient series. In this view, we have studied the predictive and prognostic interactions between T2D, HT, BMI, and *p53* status in mCRC.

Here, we show the clinical and prognostic impact of HT, T2D, and BMI in a series of mCRC patients treated in a single, high-volume, and academic institution as well as the mutational landscape of p53 in a selected group of patients.

## Patients and methods

### Patients’ selection and management

The prognostic and predictive roles of HT, T2D, and BMI in a series of 244 consecutive mCRC patients was analyzed. Patients were treated and monitored according to ESMO (European Society of Medical Oncology) guidelines (31). Patients with oligo-metastatic disease at diagnosis (one to three lesions per organ with a maximum tumor diameter smaller than 70 mm and no single lesions more than 25 mm diameter) (32, 33), age > 80 years, PS ECOG (Performance Status Eastern Cooperative Oncology Group) ≥ 2, cachexia risk > 1 (34), and life expectancy < 3 months were excluded to avoid prognostic (reasonably positive or negative) interferences. Patients were monitored with tbCT (total body Computed Tomography) scan every three months. RECIST (Response Evaluation Criteria In Solid Tumours v1.1) (35) were used to classify responses to treatment and assess disease course. In brief, CR (Complete Response) was defined as complete remission of all lesions detectable on tbCT. PR (Partial Response) was defined as at least a 30% reduction in the sum of target lesions diameters. PD (Progressive Disease) was the increase in the sum of target lesions diameters of at least 20%. SD (Stable Disease) included all changes between 20% growth and 30% decrease. DC (Disease Control) was the sum of CR + PR + SD. The study was conducted according to the standards of the Declaration of Helsinki and all patients gave a signed informed consent before administration of any treatments.

### Diabetes, hypertension and obesity definitions

Patients classified as T2D were initially diagnosed according to the American Diabetes Association criteria (casual plasma glucose concentration ≥ 200 mg/dl or fasting plasma glucose ≥ 126 mg/dl or 2-h glucose ≥ 200 mg/dl after Oral Glucose Tolerance Test) (36, 37) and they did not require insulin administration. HT was classified according to the ACC (American College of Cardiology)/AHA (American Heart Association) guidelines: normal blood pressure, less than 120/80 mm Hg; elevated blood pressure: systolic between 120 and 129 and diastolic less than 80; stage 1 HT: systolic between 130 and 139 or diastolic between 80 and 89; stage 2 HT: systolic at least 140 or diastolic at least 90 mm Hg. The hypertensive crisis was defined as a systolic pressure over 180 and/or diastolic over 120, with patients needing medications (38). Bevacizumab-induced hypertension (BIH) in subjects without HT was defined as a persistent (≥24 h) increase of systolic (140–159 mm Hg) or diastolic (90–99 mm Hg) blood pressure independently from the start of medical intervention. In patients with previous HT, it was defined as symptomatic increase by >20 mm Hg (diastolic) or to >140/90 mm Hg requiring a change in baseline medical intervention. Body mass index (BMI) was calculated through the body mass (kg) divided by the square of the body height (m). It was used to differentiate normal (18.5–24.9 kg/m^2^), overweight (25–29.9 kg/m^2^), and obese individuals (>30.0 kg/m^2^) according to WHO (World Health Organization) (39).

### NGS (next-generation sequencing) assessments

Formalin-fixed and paraffin-embedded (FFPE) tissue specimens of tumors (biopsies or resected lesions) were collected. All patients agreed and signed an informed consent to perform NGS assessments of their cancers. Tumors microdissections under morphological control was performed for each tissue specimen to isolate and enrich cancer cells. The MGF03-Genomic DNA FFPE One-Step Kit, according to the manufacturer’s protocol (MagCoreDiatech, Diatech Lab Line, Jesi, Italy), was used for DNA isolation. DNA quality was assessed using the FFPE QC Kit (Illumina, San Diego, CA, USA). TruSigt™Oncology 500 kit was applied to construct the genomic libraries; it is a comprehensive genomic profiling test targeting 523 cancer-relevant genes. The assay is able to detect indels, small nucleotide variants (SNVs), splice variants, copy-number/structural variations, and gene fusions. The present study focused on p53 gene mutations. Mutations are reported with a standard nomenclature based on reporting protein-level amino acid sequence changes with the prefixe “p.”. The Illumina NovaSeq 6000 technological platform was used for sequencing. The Illumina TruSight Oncology 500 bioinformatics pipeline as previously reported (40) was used to analyze and interpret sequencing results of this study.

### Statistical analyses and data presentation

The primary outcome of this study is the prognostic impact of T2D, BMI, and HT on overall survival (OS) of mCRC patients treated in a real practice setting. The OS was assessed from the diagnosis of metastatic disease until death from CRC (cancer-specific survival). Progression-free survival (PFS) was not included as a study objective because treatments and radiologic evaluations were heterogeneous, not centralized and the vital status is a more reliable and solid outcome to report and analyze.

To avoid statistical fragmentation and the detrimental effects on results’ interpretation of “multiplicity,” only three additional analyses were pre-planned: the studies of associations between (1) T2D, BMI, HT, and response to therapy, (2) BIH and response to anti-angiogenic therapy, (3) T2D, BMI, HT and *p53* mutations.

Data were extracted from an electronic database registering clinical and pathological characteristics of metastatic CRC patients treated at the unit of Innovative Therapies for Abdominal Metastases of the Istituto Nazionale Tumori di Napoli, “G. Pascale” foundation. In order to avoid any prognostic interferences (therapeutic, diagnostic and/or methodologic) due to physiologic evolutions occurring over time in clinical practice, the enrollment time was limited to the last six years. In univariate and multivariate analyses, putative prognostic factors (covariates) were dichotomized: gender (male vs. female), age (<65 vs. >65 years), response to first-line chemotherapy (disease control vs. no diseases control), KRAS mutations (non-mutated vs. mutated), side (left vs. right), number of metastatic sites (1 site vs. >1), T2D vs. non-T2D, blood pressure (normal blood pressure vs. systolic between 130 and 139 or diastolic between 80 and 89 mm Hg), weight (BMI <25 vs. ≥25 kg/m^2^).

OS was generated through the Kaplan–Meier product limit method. Statistical significance at univariate analysis was evaluated with a two-tailed log-rank test. Multivariate analysis was applied to study prognostic interactions between OS and covariates; the test was based on the Cox proportional-hazards regression model. The estimates of the survival probability according to different covariates were expressed through the HRs (Hazard Ratios) which are the risk of event (death), at any time, for a patient with the risk factor present compared to a patient with the risk factor absent (given both patients identical for all other covariates). HRs were reported in any analyses with 95% confidence intervals (CI). Statistical analyses were done using the Excel software and MedCalc^®^ version 20.112. Associations between HT, T2D, BMI, and clinical variables (see above) were depicted through contingency tables and evaluated by χ^2^ test. *P* < 0.05 was considered statistically significant.

## Results

### Clinico-pathological characteristics

The clinical and pathological characteristics of the analyzed clinical cohort are reported in Table 1. Two-hundred-forty-four patients were enrolled. The median age was 64 years (range: 28–79). Genders were almost equally distributed. Left sided and G3 tumors were predominant, 61.9 and 83.2%, respectively. At first diagnosis of colorectal cancer (CRC), primary tumors were mostly pT3 (68.2%). Lymph node involvement at initial staging was slightly prevalent (54.9%). One-hundred-sixty-three patients (66.8%) presented with two or more metastatic sites. One-hundred-thirty-four (54.9%) and 15 (6.1%) patients had *RAS* or *BRAF* (p.Val600Glu) mutations, respectively. One-hundred-twenty (49.2%), 110 (45.1%), and 50 (20.5%) patients were affected by overweight, HT, and T2D, respectively, at diagnosis of metastatic disease. The largest part of patients (206 patients, 84.4%) received more than two treatment lines. Almost half of patients had received adjuvant fluoropyrimidines-based therapy before developing metastatic disease. The use of chemotherapy and biologic treatments (anti-VEGF or anti-EGFR) as first-line treatment was predominant (89.6%).

**TABLE 1:** Clinico-pathological characteristics of patients.

Variable	No.	%
**Age**		
Median, range (year)	64, 28-79	
**Gender**		
Male	127	52.1
Female	117	47.9
**Side**		
Left	151	61.9
Right	93	38.1
**Grading**		
G1/G2	41	16.8
G3	203	83.2
**Lymphnodal involvement^a^**		
No	86	35.2
Yes	134	54.9
Unknown	24	9.8
**pT^a^**		
T1/T2	43	19.6
T3	150	68.2
T4	27	12.3
Unknown	24	9.8
**No. of metastatic sites**		
1	81	33.2
≥2	163	66.8
***RAS (K or N)***		
Mutated	134	54.9
Wild-type	110	45.1
***BRAF***		
Mutated	15	6.1
Wild-type	229	93.9
**Body-mass index**		
Normal	124	50.8
25–29.9 kg/m^2^ (overweight)	59	24.2
>30.0 kg/m^2^ (obese)	61	25.0
**Type 2 diabetes**		
Yes	50	20.5
No	194	79.5
**Hypertension**		
Yes	110	45.1
No	134	54.9
**No. of treatment lines**		
1	12	4.9
2	26	10.7
>2	206	84.4
**Previous adjuvant therapy**		
Yes	126	51.6
No	118	48.4
**Type of treatment**		
Only CT	29	11.9
CT plus anti-VEGF	106	43.3
CT plus anti-EGFR	109	44.7

^a^The sum of cases does not correspond to the total number of patients because some of them (24) did not receive surgical removal of primary tumor.

### Sensitivity to first-line chemotherapy in overweight, diabetic, and hypertensive patients

Microenvironment and genetic characteristics of CRC (notably associated with response to oncologic treatments) could be influenced by components of the “metabolic syndrome.” Therefore, we focused on potential associations between BMI, T2D, HT, and response to first-line treatments; subsequent therapy lines were not analyzed for the existence of too many biological and clinical biases potentially confounding the real associations in this therapeutic context. Interestingly, disease control was achieved more frequently in patients without T2D (83.1%) compared to the diabetic ones (16.9%) (*P* = 0.0246) (Table 2). Bevacizumab-induced hypertension (BIH) has been studied as a surrogate of response to bevacizumab-based therapy in CRC. On this light, we explored any correlation between BIH and disease control. Interestingly, disease control was documented in 94.3% of patients experiencing BIH versus 66.2% in patients not experiencing BIH (+28.1%; *P* = 0.0018) (Table 3).

**TABLE 2:** Associations between BMI, T2D, HT, and response to first-line therapy.

	Type of response to first-line therapy		
**Characteristic**	**No DC (%)^a^**	**DC (%)^a^**	***P***
**BMI**			
Normal	28 (45.2)	79 (51.3)	
≥25 kg/m^2^	34 (54.8)	75 (48.7)	0.4155
**T2D**			
No	43 (69.4)	128 (83.1)	
Yes	19 (30.6)	26 (16.9)	0.0246
**HT**			
No	29 (46.8)	87 (56.5)	
Yes	33 (53.2)	67 (43.5)	0.1960
**T2D, BMI, HT**			
No	48 (77.4)	130 (84.4)	
Yes	14 (22.6)	24 (15.6)	0.2229

BMI, body mass index; DC, disease control; HT, hypertension; T2D, type 2 diabetes.

^a^The sum does not correspond to the total number of patients because some of them (28) did not undergo to radiologic reassessment of disease. Statistical analysis was applied to cases with a certain evaluation of response according to RECIST v1.1.

**TABLE 3:** Association between BIH (Bevacizumab-Induced Hypertension) and first-line bevacizumab-based chemotherapy.

	BIH		
	**No (%)**	**Yes (%)**	***P***
**Disease control**			
No	22 (33.8)	2 (5.7)	
Yes	43 (66.2)	33 (94.3)	0.0018

### Prognostic power of BMI, T2D, and HT in metastatic CRC

**FIGURE 1:**
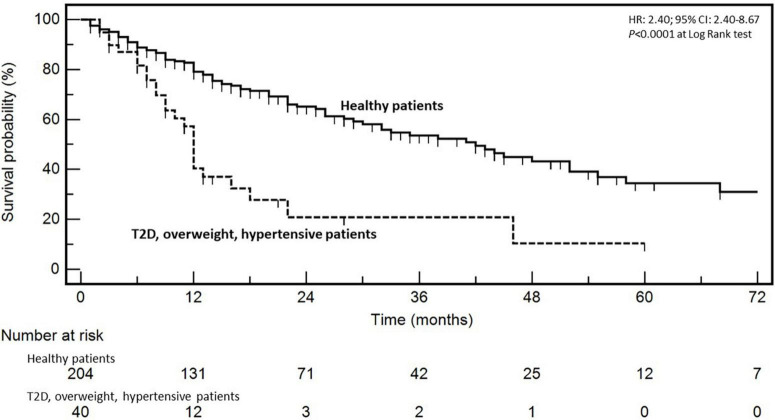
Kaplan–Meyer survival curves according to the presence of type 2 diabetes, body-mass index ≥25 mg/m^2^ and hypertension in metastatic colorectal cancer patients (healthy patients are those without diabetes, hypertension, and overweight). Hazard ratios (HR), confidence intervals (CI), and *P* at log-rank test are embedded in the figure.

In order to confirm the impact on patient clinical outcome, the prognostic value of BMI, T2D, and HT was analyzed by means of both uni- and multivariate analyses (Table 4). The median follow-up for the entire cohort was 46.0 months; at the time of the analysis, 110 cancer-specific deaths were recorded. Response to first-line chemotherapy (DC vs. no DC), *KRAS* mutational status (mutated vs. wild-type), T2D (present vs. not present), BMI (>25 kg/m^2^ vs. normal), and concomitant presence of T2D, BMI, and HT associated with survival at univariate analysis (Table 4). At multivariate analysis, age (≥65 vs. <65 years; HR: 1.44; 95% CI: 0.97–2.14; *P* = 0.0290), response to first-line chemotherapy (DC vs. no DC; HR: 0.34; 95% CI: 0.20–0.56; *P* < 0.0001), and concomitant presence of T2D, BMI, and HT (HR: 4.56; 95% CI: 2.40–8.67; *P* = 0.0217) emerged as independent prognostic variables (Table 4). Survival of patients according to the concomitant presence of T2D, BMI, and HT is depicted in Figure 1.

**TABLE 4:** Uni- and multivariate analysis of prognostic power of clinical characteristics and metabolic syndrome components in metastatic CRC patients.

Co-variate	Dicothomization	Median survivals (months)	No. of events/Patients	*P* at univariate	HR	95% CI	*P* at multivariate
Age	≥65 vs. <65 years	26.0 vs. 46.0	79/157 vs. 31/87	0.0641	1.44	0.97–2.14	0.0290
Gender	M vs. F	44.0 vs. 30.0	50/117 vs. 60/127	0.5736	0.89	0.61–1.31	0.7122
Side	L vs. R	46.0 vs. 32.0	70/151 vs. 40/93	0.2113	1.27	0.86–1.88	0.3271
Metastatic involvment	>1 vs. 1 site	33.0 vs. 28.0	44/81 vs. 66/163	0.0875	1.42	0.94–2.13	0.7863
Response to first-line CT	DC vs. no DC	46.0 vs. 15.0	62/154 vs. 36/62	<0.0001	0.34	0.20–0.56	<0.0001
KRAS mutations	Mutated vs. wild-type	30.0 vs. 42.0	65/134 vs. 45/110	0.0495	1.46	1.00–2.13	0.2993
T2D	Present vs. not present	12.0 vs. 42.0	32/50 vs. 78/194	0.0001	2.91	1.72–4.94	0.4165
BMI	≥25 kg/m^2^ vs. normal	22.0 vs. 45.0	64/120 vs. 46/124	0.0040	1.75	1.19–2.58	0.2054
HT	Present vs. not present	26.0 vs. 42.0	57/110 vs. 53/134	0.0838	1.40	0.95–2.06	0.5681
Cluster of T2D, BMI, HT	Present vs. not present	12.0 vs. 42.0	25/40 vs. 85/204	<0.0001	4.56	2.40–8.67	0.0217

CI, confidence interval; DC, disease control; F, female; HR, hazard ratio; L, left; M, male; R, right.

### *P53* mutational landscape: Association with BMI, T2D, HT

**FIGURE 2:**
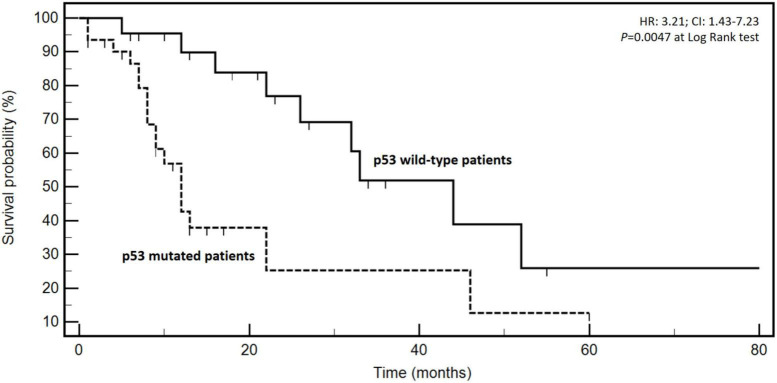
Kaplan–Meyer survival curves according to the presence or not of p53 mutations. Hazard ratios (HR), confidence intervals (CI), and *P* at log-rank test are embedded in the figure.

Since *p53* has been implicated in cancer metabolism reprogramming and several metabolic disorders including T2D and obesity, we studied the mutational landscape of the protein in a group of 53 patients of our series in order to explore any associations with BMI, T2D, and HT. *P53* was mutated in 31 out of 53 cases (58.5%). Clinico-pathological characteristics of this clinical subgroup are reported in Table 5. The most frequent alterations were p.Arg175His and p.Cys135Tyr (Table 6). Interestingly, a strong association was found between high BMI (>25 kg/m^2^) and the occurrence of *p53* mutations (*P* < 0.0001) (Table 7). No relationships emerged with T2D and HT. The association of high BMI, T2D, and HT with *P53* alteration (*P* = 0.0293) was reasonably very likely attributable to the presence of overweight patients. *P53* mutations have been described to have a negative prognostic impact in CRC. Therefore, the prognostic role of *p53* mutations was analyzed in our cohort and depicted in Figure 2. Interestingly, *p53* mutated patients presented a worse prognosis (median OS: 12.0 months) compared to the wild-type ones (median OS: 44.0 months) (events: 18/31 vs. 9/22; HR: 3.21; 95% CI: 1.43–7.23; *P* = 0.0047) (Figure 2). The combination of BMI >25 kg/m^2^ and presence of *p53* mutation even reinforced the negative prognostic impact (median OSs: 11.0 vs. 46.0 months; events: 17/27 vs. 10/26; HR: 3.62; 95% CI: 1.33–7.78; *P* = 0.0032).

**TABLE 5:** Clinico-pathological characteristics of p53 assessed patients.

Variable	No.	%
**Age**		
Median, range (year)	62, 32–70	
**Gender**		
Male	34	64.1
Female	19	35.9
**Side**		
Left	31	58.5
Right	22	41.5
**Grading**		
G1/G2	10	18.9
G3	43	81.1
**Lymphnodal involvement^a^**		
No	24	45.3
Yes	23	43.3
Unknown	6	11.4
**pT^a^**		
T1/T2	14	26.4
T3	27	50.9
T4	6	11.3
Unknown	6	11.3
**No. of metastatic sites**		
1	31	58.5
≥2	22	41.5
***RAS (K or N)***		
Mutated	30	56.6
Wild-type	23	43.4
***BRAF***		
Mutated	5	9.4
Wild-type	48	90.6
**Previous adjuvant therapy**		
Yes	22	41.5
No	31	58.5
**No. of treatment lines**		
1	5	9.4
2	6	11.3
>2	42	79.2
**Response to first-line CT^b^**		
DC	34	64.1
No DC	14	26.4
Not assessed	5	9.4

CT, chemotherapy; DC, disease control.

^a^The sum of cases does not correspond to the total number of patients because some of them (6) did not receive surgical removal of primary tumor.

^b^The sum does not correspond to the total number of patients because some of them (5) did not undergo to radiologic reassessment of disease.

**TABLE 6:** *p53* mutational landscape.

p53 mutational status	No.	ClinVar ID	Functional consequence
Wild-type	22		
Mutated	31		
p.Arg175His	4	12374	GOF
p.Cys135Tyr	4	141762	LOF
p.Gln100Ter and p.Pro72Arg	2	634707	LOF
p.Glu286Lys and p.Pro72Arg	1	183752	LOF
p.Arg273His	1	12366	GOF
p.Val173Ala	1	376017	LOF
p.Arg306Ter and Pro72Arg	1	142144	LOF
p.Arg65Ter and p.Pro72Arg	1	634670	LOF
p.Arg213Ter	1	43590	LOF
p.Arg175His and p.Pro72Arg	1	12374	LOF
p.Arg306Ter	1	142144	LOF
p.Arg196Ter	1	43589	LOF
p.Arg248Trp and p.Pro72Arg	1	12347	GOF
p.Arg273Cys	1	43594	LOF
p.Arg248Gln	1	12356	GOF
p.Cys38Phe and p.Pro72Arg	1	Not present	Not reported
p.Cys135Phe	1	376559	LOF
p.Gly245Ser	1	12365	GOF
p.Gly108ValfsTer15	1	Not present	Not reported
p.Gly244Cys	1	376599	LOF
p.Ile195Thr	1	216077	LOF
p.Pro152Leu	1	142766	LOF
p.Ser24Phe	1	Not present	Not reported
p.Tyr220Cys	1	127819	LOF

GOF, gain of function; LOS, loss of function; Ter, termination codon.

**TABLE 7:** Associations between BMI, T2D, hypertension, and p53 mutational status.

	p53 mutational status		
**Characteristic**	**Wild-type (%)**	**Mutated (%)**	***P***
**BMI**			
Normal	17 (77.3)	4 (12.9)	
≥25 kg/m^2^	5 (22.7)	27 (87.1)	<0.0001
**T2D**			
No	18 (81.8)	20 (64.5)	
Yes	4 (18.2)	11 (35.5)	0.1723
**Hypertension**			
No	13 (59.1)	17 (54.8)	
Yes	9 (40.9)	14 (45.2)	0.7605
**T2D, BMI, HT**			
No	20 (90.9)	20 (64.5)	
Yes	2 (9.1)	11 (35.5)	0.0293

BMI, body mass index; T2D, type 2 diabetes.

## Discussion

Nowadays, 39% of adults of the worldwide populations is overweight; 13% is obese (41), and, unfortunately, the global prevalence of overweight and obese individuals is tripled since 1975. Even more frequent (about 40%) in Western adults is HT. On the other hand, CRC is the third most common cancer in both genders and the concomitant presence of CRC, obesity, T2D, and HT in real practice is frequently observed. Interestingly, a cause-effect relationship between obesity, T2D, HT and CRC have been argued in about half of cases. Thus, the biologic and prognostic connections are reasonably profound and still not completely explored.

In our study, we focused on the prognostic and predictive interconnections between BMI, T2D, and HT in a large series of mCRC patients.

Interestingly, we found that diabetic patients are less prone to respond to first-line chemotherapy and they have a worse prognosis compared to not diabetic ones. This was not related to reduced dose intensity in this clinical series (Supplementary file). On a biologic point of view, the insulin resistance [due to hyperinsulinemia, increased levels of Insulin-like Growth Factors (IGFs), and hyperglycemia] (42, 43) and the chronic inflammatory status (due to pro-oxidative status and enhanced generation of reactive oxygen species) (44–47) may account for both unresponsiveness and unfavorable prognosis of these patients. A complete dissertation about the biological factors involved in the interrelationship between cancer progression and T2D is beyond the scope of this discussion. Noteworthy, in the present manuscript we did not measure IGFs and insulin. This is a limitation of our study that prevents us from hypothesizing a direct mechanistic correlation between cancer, overweight and diabetes. However, C-reactive protein (CRP) levels were available in 197 out of 244 patients before starting treatments. CRP indicates a chronic subclinical inflammatory status (48, 49) and was significantly higher in T2D (42 cases) vs. not T2D (155 cases) patients in our series (means ±2 standard deviations: 5.8 ±1.3 vs. 3.2 ±1.6 mg/L, *P* < 0.05 with the *t*-test). Mechanistically, a number of molecular pathways involved in cancer progression are also altered in chronic inflammation and insulin resistance frequently present in T2D and/or overweight patients (AMPK, adenosine monophosphate-activated protein kinase; CDKs, Cyclin-dependent kinases; HDAC, histone deacetylase; IGFs; PDH pyruvate dehydrogenase complex; PI3K/Akt, phosphatidylinositol 3-kinase/protein kinase B; SGLT2, sodium glucose co-transporter 2; STAT1, signal transducer and activator of transcription 1; wnt, wingless/integrated) (44–49). To this regard, recent advances on the biology of dysmetabolic mCRC patients (50–55) indicate that therapeutic strategies aiming to impact on insulin resistance and chronic inflammation, in addition to improving glycemic control, could reasonably restore sensitivity to chemotherapy and ameliorate the cancer-specific survival.

Interestingly, all the T2D patients included in this study received metformin. The use of this drug (an oral lipophilic biguanide) is a standard first-line therapy (along with lifestyle changes) for the glycemic control in T2D patients. Notably, metformin has demonstrated anti-cancer properties through several mechanisms including direct stimulation of apoptosis, potentiation of the immune system, sensitization of cancer cells to chemotherapy, inhibition of oxidative stress and inflammatory cancer-supporting pathways (56). Unfortunately, the use of metformin was not associated with improved survival in this study. To this regard, the mean glycemia value in our T2D patients at diagnosis was 159 mg/dl (95% CI: 125–182 mg/dl) indicating a scarce control of glycemia (57). Therefore, a direct effect of glucose on worsening the prognosis of our patients and contributing to decrease responsiveness to chemotherapy cannot be excluded. A poor glycemic control could explain the lack of anti-cancer effects of metformin in our series.

Noteworthy, we failed to demonstrate nor a predictive (*P* = 0.1960) neither a prognostic (*P* = 0.0838) role for HT in our cohort. This could be related to the high incidence of HT in our population as well as to a selection bias related to the heterogeneity of pressure measurements and reporting, and to the retrospective nature of our cohort. By contrast, a significant correlation was revealed in patients experiencing BIH with response to bevacizumab-based first-line chemotherapy, as also previously reported (58).

*P53* is the most altered gene in cancer and has very pleomorphic roles in both physiological and pathological conditions (22). Furthermore, it is a paradigm of tumor biologic and genetic complexity since its mutations can lead to loss or gain of function (respectively LOF and GOF mutations) with differential clinical and prognostic consequences (59). In particular, *p53* has been implicated in cancer metabolism reprogramming (60) and in lipid and glucose homeostasis controlling the expression of proteins regulating glycolysis, glucose transport, lipid absorption, transport, biosynthesis, and desaturation (61). In physiologic conditions, the activation of transcriptional activity of *p53* produces negative effects on glycolysis and adipocytes while most *p53* mutations are associated with hyperglycemia and adipocyte hyperplasia (62). Very few background data were previously reported and, therefore, in the present study an exploratory and hypothesis-generating analysis of the interaction between *p53* mutations, T2D, BMI, and HT was performed. It has been previously reported a prognostic role for *p53* in metastatic CRC patients treated with chemotherapy (63). Therefore, a limited sample size was genetically characterized. We found a strong association between the presence of *p53* mutations and BMI ≥ 25 kg/m^2^ (indicating overweight/obese patients). These results, although interesting, must be interpreted with caution considering that not all patients’ specimens were available for NGS assessment because of technical (DNA quality) and patients-related reasons (no consent or death). Therefore, the data obtained are limited by the extent of the sample size, the possible selection bias and the heterogeneous nature of the revealed *p53* mutations, even if the last was frequently reported. Considering the surprisingly strong association between *p53* and BMI, it should be interesting to evaluate if combinations of different BMI values with the presence of *p53* mutations ameliorated the prognostic power. High BMI and *p53* mutated tumors, although not tested in multivariate analysis to avoid multiplicity and spurious results, emerged as a promising prognostic subgroup to validate in prospective and larger series. Another limitation of our study, in addition to its retrospective nature, is the absence of lipids level report. Lipids dysregulation is one of the main components of the “metabolic syndrome”. Therefore, these data can add insights and information on the prognostic role of metabolic disorder in this cohort of mCRC patients.

In conclusion, T2D patients are less prone to respond to chemotherapy and the cluster of T2D, BMI, and HT emerges as an independent prognostic factor in mCRC patients. A personalized therapeutic approach, rather than chemotherapy potentiation, is needed in diabetic, overweight, and hypertensive mCRC patients.

## Data availability statement

The datasets presented in this study can be found in online repositories. The names of the repository/repositories and accession number(s) can be found below: European Nucleotide Archive (ENA), under accession PRJEB58429.

## Ethics statement

Ethical review and approval was not required for the study on human participants in accordance with the local legislation and institutional requirements. The participants of this study provided a written informed consent before any treatments and/or genetic assessments.

## Author contributions

AO, MSa, GS, and MCar: conceptualization. AO, MCar, FP, VG: methodology. MSa and MCasc: software. FS and OG: validation. AO, MCar, and MCap: formal analysis. LC, MI, MCasi, AL, and NP: investigation. AO and GN: resources. AO, MSc, AL, SZ, and VG: data curation. AO, MS, and MCar: writing—original draft preparation. GN, GS, FP, and FS: writing—review and editing. AO, MCar, and GS: supervision. All authors contributed to the article and approved the submitted version.
