# Supplementary material for: Hypertension, type 2 diabetes, obesity, and p53 mutations negatively correlate with metastatic colorectal cancer patients’ survival
Source: Front Med (Lausanne). 2023 Jan 23;10:1091634. doi: 10.3389/fmed.2023.1091634 (PMC9899824; doi:10.3389/fmed.2023.1091634)
Supplement: Supplementary file 1 [file Data_Sheet_1.PDF]

Dose intensity of first-line chemotherapy in presence of high BMI, T2D and HT.

	Dose delays ≥ 7 days		
	**No (%*)**	**Yes (%)**	***P***
**High BMI/T2D/HT**			
**Yes**	29 (72.5)	11 (27.5)	
**No**	159 (77.9)	45 (22.1)	0.4552
	**Dose reductions ≥ 25%**		
**High BMI/T2D/HT**	**No (%)**	**Yes (%)**	***P***
**Yes**	24 (60.0)	16 (40.0)	
**No**	138 (67.6)	66 (32.4)	0.3502

High BMI: Body Mass Index ≥25 kg/m^2; T2D: Type 2 Diabetes; HT: Hypertension.
*Row percentages.
P at chi-square test.
